# Editorial: International Women of Supramolecular Chemistry

**DOI:** 10.3389/fchem.2022.854085

**Published:** 2022-02-28

**Authors:** Claudia Caltagirone, Emily R. Draper, Jennifer S. Leigh, Cally J. E. Haynes, Jennifer R. Hiscock, Anna J. McConnell

**Affiliations:** ^1^ Department of Chemical and Geological Science, University of Cagliari, Monserrato, Italy; ^2^ School of Chemistry, University of Glasgow, Glasgow, United Kingdom; ^3^ Centre for the Study of Higher Education, University of Kent, Canterbury, United Kingdom; ^4^ Department of Chemistry, University College London, London, United Kingdom; ^5^ School of Chemistry and Forensics, University of Kent, Canterbury, United Kingdom; ^6^ Otto Diels Institute of Organic Chemistry, Christian-Albrechts-Universität zu Kiel, Kiel, Germany

**Keywords:** supramolecular chemistry, women in chemistry, equality, diversity, inclusion

There is a need to address the lack of diversity in Chemistry (Urbina-Blanco et al., 2020). Despite dedicated programmes and outreach activities to attract individuals from minority backgrounds to initiate their academic studies in this area, at the most senior levels there remains an underrepresentation of minority and marginalised groups (RSC, 2018). People who have one or more protected EDI (Equality, Diversity, Inclusion) characteristic such as race, religion, disability, sexuality, or gender face more barriers to remain and to succeed within science.

The International Women In Supramolecular Chemistry (WISC) network, has taken a unique, area-specific approach that embeds EDI and creative social sciences to address the retention and progression of women and other marginalised groups (Caltagirone et al., 2021).

Our community-led approach to researching *with*, and **NOT** *on* scientists allows us to shape our offering to the community to specifically address its needs. In response to our first survey of the community, we created a mentoring programme, a series of webinars (in collaboration with vMASC—the Early Career arm of the Royal Society of Chemistry (RSC) Macrocyclic and Supramolecular Chemistry Special Interest Group), a series of virtual spaces where individuals of any gender could come together to find support (termed Community Clusters), and a Skills Workshop. These all sought to address the feelings of isolation and the progression gap shared by many respondents as they transitioned post-PhD to a post-doc/PDRA, fellowship, or related industrial position.

The mentoring programme run by WISC uses a model whereby small groups of up to five individuals at a similar career stage meet regularly with a mentor who is at least one career stage ahead of them. In the first 18 months over 90% of participants reported being satisfied with the programme and wishing to continue. This was an initiative that the supramolecular community asked WISC to initiate and is further known in literature to be particularly effective at supporting those who are marginalised (Laube and Crimmins, 2019).

The WISC collaboration with vMASC has been very successful, with seminars organised on topics such as careers outside of academia, science communication, and work-life balance attracting a wide audience across Europe, the United Kingdom, US, India, and Australia. In addition, vMASC provided the technical support to make the first WISC Skills Workshop (2021) a hybrid in person/virtual event, with over 200 registrations. One attendee said “*I liked the informative style, the small nature of the workshop group and the fact that almost everyone on site had the chance to participate (oral or 3 *min *poster presentation). I found the workshop to be extremely informative. I especially liked the way in which it encouraged the early career researchers to think outside the box.*”

WISC takes an intersectional approach to marginalisation, recognising that not all groups face the same barriers, and that those who have more than one protected characteristic face compounded barriers to reach their full potential. In response to this need, we initiated our virtual “Community Clusters” to provide safer spaces and dedicated support. The first cluster was the “Parenting Cluster” which aimed to support all people on a parenting journey—whether they were prospective parents, foster parents, adoptive parents, or step-parents. The second cluster was for disabled/neurodivergent/chronically ill people. This cluster provides a regular meeting space for people to talk and share experiences, and recently, gained RSC support for a project to envision future accessible labs. Our final cluster is the “first Gen Cluster” which will specifically look to support those who are first in their family into higher education within supramolecular chemistry.

WISC also undertakes high-quality qualitative research to capture the voices and stories of women and other marginalised groups.^1^ We intentionally use a variety of creative and more traditional methods as part of an Embodied Inquiry (Leigh and Brown, 2021) that foregrounds lived experiences. During COVID-19 we triangulated a qualitative survey, a collaborative autoethnography, and reflective research group meetings to gather data on experiences both inside and outside the lab (Leigh et al., 2022). From these data we created a series of fictional vignettes to illustrate the lived experiences of women within supramolecular chemistry which will be published in the forthcoming book from Policy Press *Women in Supramolecular Chemistry: Collectively crafting the rhythms of our work and lives in STEM* (Leigh et al., 2022). Our current research includes looking at how creativity and communication can be improved within research teams led by women, and their role in developing leadership skills. We have associated outreach and Public Engagement projects, including a collaboration with Empowering Female Minds in STEM, (EFeMS, 2021) to increase the visibility of Black women in chemistry and science communication, and the National Association for Disabled Staff Networks STEMM Action Group to provide recommendations for funders and institutions to support disabled scientists.

This Special Issue, with articles showcasing the work of women in supramolecular chemistry, is part of our commitment to action and change. Women face barriers at every stage of the publication process (RSC, 2019). Bringing their work together allows the supramolecular chemistry community an opportunity to see and value the ground-breaking science they are achieving despite the additional barriers they face just because of their gender. Bringing their work together allows the community to recognise and celebrate them. It will allow those coming through from undergraduate study to see that it is possible for women and other marginalised groups to succeed, and that there is a place for them.

WISC chooses not to “call out” instances of sexism or misogyny. Instead, we “call in” the community so that it can support its own (WISC, 2021). In this way we can create a supportive and inclusive environment which holistically enables the retention and progression of everyone—regardless of gender, race, disability, or any other characteristic. We need to continue the successful outreach that invites more diversity in chemists from undergraduate level upwards, and we need to support their retention and progression so that they can see that there is a clear path forward for everyone.

WISC has big aspirations and ambitions for the supramolecular community. We would like the model we have created and the work that we are doing to act as a framework for other area-specific networks and disciplines, reaching out to other marginalised communities. In order to do this, we need the community to be involved—so please get in touch via www.womeninsuprachem.com if you:

• would like to be a mentor, or a mentee (or both)
• would like to be part of one of our virtual Community Clusters or help us to create a new one;
• would like to be part of our research, or to find out more about what we are doing to support women, disabled scientists, and the visibility of all women in science communication.

## Footnotes

1 WISC believes that trans women are women. The use of other marginalised groups specifically includes other marginalised genders including trans men and non-binary people as well as those marginalised due to one or more other protected characteristic such as race, ethnicity, religion, sexuality, or disability
